# Saikosaponin-b2 Regulates the Proliferation and Apoptosis of Liver Cancer Cells by Targeting the MACC1/c-Met/Akt Signalling Pathway

**DOI:** 10.1155/2024/2653426

**Published:** 2024-11-06

**Authors:** Yanxue Zhu, Xingzhi Lv, Ruifang Li, Zihan Gao, Chanhao Lei, Lan Wang, Sanqiang Li

**Affiliations:** Department of Pharmacology, Basic Medicine and Forensic Medicine College, Henan University of Science and Technology, KaiYuan Road 263, Luoyang 471023, Henan, China

**Keywords:** c-Met signalling, liver cancer, metastasis-associated in colon cancer-1, saikosaponin-b2

## Abstract

Saikosaponin-b2 (SS-b2), an active ingredient derived from the root of Radix Bupleuri, possesses antitumour, anti-inflammatory, antioxidative and hepatoprotective properties. We investigated the inhibition of tumour proliferation by SS-b2 and the underlying molecular mechanisms, including the MACC1/p-c-Met/p-Akt pathway expression in HepG2 liver cancer cells and H22 tumour-bearing mice. Animal experiments showed that SS-b2 significantly decreased the levels of MACC1, p-c-MET and p-Akt in tumour tissue transplanted with H22 liver cancer cells in mice, while it increased the expression of p-BAD. The results also revealed a concentration-dependent suppression of MACC1, p-c-Met and p-Akt expression in the SS-b2 treatment group compared with the control group. Additionally, the suppression of MACC1 activation by SS-b2 resulted in a reduction in the viability and proliferation of HepG2 liver cancer cells, and this reduction was comparable to that by doxorubicin (DOX). This suggests that SS-b2 has significant efficacy in liver cancer, comparable to DOX. Meanwhile, Annexin V-FITC/PI staining and western blot analysis of cleaved caspase 9 and cleaved caspase 3 demonstrated that SS-b2 induced apoptosis of HepG2 liver cancer cells. These findings provide experimental evidence suggesting that SS-b2 is a promising anticancer agent for liver cancer.

## 1. Introduction

Liver cancer is a highly prevalent malignant tumour globally, with a particularly elevated incidence and mortality in Asia. Nevertheless, there has been a notable increase in the prevalence of liver cancer in other regions worldwide as well. Because the onset of liver cancer is occult, most liver cancer cases are in the final stages at diagnosis, and to date, no completely effective treatment is available for liver cancer. Over the past two decades, research interest in natural products as a valuable source of small-molecule drugs has increased [1]. Saikosaponins are the main bioactive ingredients of the traditional Chinese medicine Radix Bupleuri and exhibit antitumour, antioxidative damage, antifibrosis and immune regulatory activities [2–4]. Research has shown that saikosaponins can prevent and treat cancer by acting through multiple routes and on multiple targets and thus have a great prospect for clinical application [5, 6]. Saikosaponin-b2 (SS-b2), a triterpenoid saponin derived from saikosaponins, has shown potent anticancer activity in liver cancer. However, its underlying mechanisms remain unclear [7, 8].

Arlt and Stein were the first to identify metastasis-associated in colon cancer-1 (MACC1) as a prognostic biomarker of cancer invasion and metastasis in human colon cancer tissue, metastatic site tissue and normal tissue [9–11]. MACC1 is a regulator of the HGF/c-Met, phosphatidylinositol 3-kinase (PI3K)/Akt and MAPK signalling pathways, which are known to play a crucial role in the growth of cancer cells. Several studies have indicated that MACC1 also plays a role in cell apoptosis [12–14].

Because the development of anticancer drugs through chemical synthesis is expensive and difficult, our group is currently focussed on finding potential anticancer drugs from natural sources such as plants and minerals or their derivatives. Discovery of anticancer drugs from plant sources has become a major area of cancer research worldwide [15]. Developing nature-origin drugs that are safe and effective with low toxicity is crucial for liver cancer treatment. Radix Bupleuri is a traditional Chinese medicine that is slightly cold in nature and tastes bitter and spicy and has therapeutic effects on the liver and gallbladder meridian [16, 17]. In accordance with the tenets of traditional Chinese medicine, it exhibits liver-protective, antiviral and antitumour effects in clinical use. The main active constituents of Radix Bupleuri are saikosaponin, essential oil and polysaccharides. Saikosaponins are divided into many subtypes, namely, saikosaponin-a (SS-a), saikosaponin-b (SS-b), saikosaponin-c (SS-c) and saikosaponin-d (SS-d), all of which are principal bioactive components [18–22]. Although SS-b2 is an SS-b, it is unknown whether SS-b2 has any effect on the proliferation and apoptosis of liver cancer cells. This study is the first to demonstrate that SS-b2 is a potent inhibitor of MACC1 and can target the c-Met/Akt signalling pathway in HepG2 liver cancer cells and H22 sarcoma xenograft mice.

## 2. Materials and Methods

### 2.1. Antibodies and Reagents

Antibodies against Bcl-2 (sc-509), Bax (sc-7480), Cyt-c (sc-13156), cleaved caspase 9 (sc-17784) and cleaved caspase 3 (sc-271759) were procured from Santa Cruz Biotechnology (CA, USA). Antibodies against MACC1 (ab106579), phosphorylated c-Met (Y1234 + Y1235) (ab5662), phosphorylated Akt (S473) (ab81283) and phosphorylated BAD (S112) (ab129192) were procured from Abcam (Cambridge, MA, UK). Antibodies against *β*-actin (60008-1-Ig), HRP-conjugated goat anti-rabbit IgG (SA00001-2) and HRP-conjugated goat anti-mouse IgG (SA00001-1) were ordered from Proteintech Group (Wuhan, China). RIPA lysis buffer (#89900), enhanced chemiluminescence (ECL) substrate (#32109), prestained protein ladder (#26616) and Hoechst 33258 dye (H3569) were acquired from Thermo Fisher Scientific (Carlsbad, CA, USA). RNAiso Plus reagent (#9108), qPCR mix and cDNA were purchased from Vazyme Biotech Co., Ltd. The FITC Annexin V apoptosis detection kit was provided by BD Biosciences (San Jose, CA, USA).

### 2.2. Chemicals and Animals

SS-b2 (molecular weight: 780.99), with a purity of more than 98%, was procured from Chengdu Mansite Bio-Technology Co. (Chengdu, China). DOX (molecular weight: 579.98), with a purity 98%–102%, was ordered from Sigma-Aldrich (St. Louis, MO, USA). For in vivo experiments, SS-b2 and DOX were solubilised in dimethyl sulfoxide (DMSO) to obtain stock concentrations of 4 mg/mL and stored at −20°C. The solutions were then diluted in normal saline to achieve the desired concentrations prior to use. For in vitro experiments, the same solutions prepared for in vivo experiments were used, except that the stock solutions were diluted in DMEM (Solarbio, Beijing, China) containing 10% (v/v) foetal bovine serum (FBS) (Biological Industries, Cromwell, CT, USA) and the final concentration of DMSO was less than 0.1% [23].

Six-week-old male BALB/c mice, with an average weight of 18–22 g, were purchased from Henan skbex Biotechnology Co., Ltd. The mice were maintained under conditions of 22°C–26°C temperature, 40%–55% humidity and a 12-h light/dark cycle and had free access to standard food and water. All animal experiments were approved by the Ethics Committee of Henan University of Science and Technology and followed Ethical Guidelines for the Use of Animals in Research [24].

### 2.3. In Vivo Tumour Xenograft Experiment

H22 liver cancer cell lines were resuscitated from cryopreservation and then diluted to a concentration of 10^7^ cells/mL and intraperitoneally injected into mice. After 7 days of injection, the ascites fluid was drawn from the H22-bearing mice under aseptic conditions and then diluted with aseptic saline to a final concentration of 10^7^ cells/mL. Trypan blue staining showed that 95% of the cells were viable. Subsequently, 0.2 mL of cells was subcutaneously inoculated into the right armpit of each mouse to establish H22 model mice. Twenty-four hours after inoculation, the mice were weighed and randomised into four groups with 10 mice in each group: control group, in which the mice were given an intraperitoneal injection of saline; DOX group, in which mice were given an intraperitoneal injection of DOX; and two SS-b2 groups, one each given 5 and 10 mg/kg SS-b2 per day by intraperitoneal injection for 14 days [25].

### 2.4. Histology and Immunohistochemical Staining

After 24 h of fixation with 4% paraformaldehyde, tumour specimens were dehydrated and subjected to paraffin embedding. Each paraffin block was cut into ∼2-*μ*m-thick sections and stained with haematoxylin and eosin (HE). In addition, the slices were immersed in a citric acid repair solution and subjected to heat treatment for the purpose of antigenic repair. After washing three times with PBS, the endogenous enzymes were inactivated with 3% hydrogen peroxide, washed with distilled water and blocked with blocking solution. The slices were then incubated with primary antibody at 20°C for 2 h, followed by washing and then incubation with the secondary antibody at room temperature for 30 min. Finally, the slices were incubated with streptavidin-POD for 30 min at room temperature and then treated with DAB for colour development [26]. Once the tissue had begun to turn yellow, the reaction was terminated with distilled water. This was followed by haematoxylin restaining, rinsing under running water to counteract the blue colour, dehydration using an alcohol gradient, sealing the sample with neutral resin and observation under a microscope.

### 2.5. Cell Lines and Culture

HepG2 liver cancer cells was acquired from the American Type Culture Collection (Manassas, VA, USA) and maintained in DMEM containing 10% FBS nutrient solution culture cell in a damp incubator at 37°C and 5% CO2. The cells were grown in a culture flask until reaching 70%–80% confluence, washed with PBS, digested with trypsin, centrifuged and then resuspended in culture medium. All experiments were performed with cells in the logarithmic phase [27].

### 2.6. Cell Viability Assay

The cells were inoculated in 96-well plates at a density of 1 × 10^5^ per well and incubated for 24 h in culture medium containing different concentrations of SS-b2 (0.800, 0.400, 0.200, 0.100, 0.075, 0.050, 0.025 and 0.010 mg/mL) with DMSO ≤ 1%. Subsequently, the cell growth was analysed. Five duplicate wells were established for each group. Following 24-h incubation, each well of the culture plate was inoculated with 10 *μ*L of the Cell Count Kit-8 (CCK-8) solution (MCE, Monmouth Junction, NJ, USA), and the plate was incubated for 20–30 min. The optical density (OD) values of the well contents were then measured at 450 nm using a microplate reader (BioTek, Winooski, VT, USA), and the half-maximal inhibitory concentration (IC_50_) was calculated [28].

### 2.7. Hoechst 33258 Staining

Cells were inoculated in 48-well plates at a density of 2 × 10^5^ cells per well, and the plates were incubated for 24 h. Once the cell growth reached 60%–80% confluence, SS-b2 (40 or 80 mg/L) or DOX (2 mg/L) was added to the wells, and the plate was incubated for another 24 h. Following a wash with PBS and fixation with 4% paraformaldehyde for 15–20 min, the cells were stained with Hoechst 33258 solution (MCE, Monmouth Junction, NJ, USA) for 5 min. Finally, the morphological changes in the cells were visualised under a fluorescence microscope [29].

### 2.8. Annexin V-FITC/Propidium Iodide (PI) Staining

The cells were seeded in 6-well plates at a density of 10 × 10^5^ cells/well and incubated overnight. The cells were further incubated with SS-b2 (40 and 80 mg/L) or DOX (2 mg/L) for 24 h. The cells were harvested, washed with precooled PBS and then stained with PI. The FITC Annexin V Apoptosis Kit was then used to detect cell apoptosis according to the manufacturer's instructions. Flow cytometry analysis was performed using the BD Accuri C6 Flow Cytometer (BD, USA), and the data were analysed using FCS Express software Version 3 [29–31].

### 2.9. Western Blotting Analysis

The tumour specimen stored at −80°C was removed and placed on ice. Thereafter, 50 mg of tissue was weighed, minced, placed in a centrifuge tube and lysed with 500 *μ*L of RIPA lysis solution on ice. The tube was centrifuged at 12,000 rpm and 4°C for 20 min, the tissue proteins were extracted and the protein concentrations were determined. Each sample contained a total of 30 *μ*g of protein, which was separated using 10% SDS-PAGE and transferred onto a PVDF membrane and blocked with milk powder for 2 h. The membrane was incubated with primary antibody overnight at 4°C, followed by incubation with the secondary antibody for 1 h at room temperature. Luminescence development was carried out using an ECL system, and band intensities were analysed using Image J [32].

The same procedure as outlined in Section 2.8 was used to treat HepG2 liver cancer cells with SS-b2 saponins and Adriamycin. Cellular proteins were extracted by lysis using RIPA lysate on ice, and protein concentrations were determined. Western blotting analysis was then carried out as described above.

### 2.10. Reverse Transcription Quantitative Polymerase Chain Reaction

The cells were treated with SS-b2 or DOX in accordance with the methodology outlined in Section 2.8. The total RNA was isolated using the TRIzol reagent in accordance with the established protocol. The quality of the extracted RNA was assessed using agarose gel electrophoresis, and the RNA concentration was measured using an RNA purity analyser (Thermo Fisher Scientific, USA). cDNA was synthesised using a reverse transcription qPCR kit and amplified using a qPCR amplification kit. The CT value and specificity of the tested genes were determined by plotting a dissolution curve of qPCR amplification products. MACC1 and c-Met gene expression levels were calculated using 18S rDNA as an internal reference gene. Primer sequences for the MACC1 and c-Met genes were designed based on their gene information in GenBank and are listed in Table 1 [33].

### 2.11. Immunocytochemistry

The slide was placed in a 6-well plate, and the cell suspension was dropped onto the slide. After the cells had adhered to the slides, the drug-containing medium was added. After the intervention was completed, the slide was removed, and the slides were washed three times with PBS and fixed with 4% paraformaldehyde for approximately 10 min at room temperature, followed by washing three times with precooled PBS. The slides were then placed in a staining tray containing antigen repair solution, heated in a 95°C water bath for 10 min and washed three times with PBS. The cells were then incubated with 0.1% Triton-X 100 for 5–10 min, washed with PBS and then incubated with 1% BSA and 22.52 mg/mL glycine in PBST for 30 min to avoid nonspecific binding of the antibody. This was followed by incubation with primary antibody overnight at 4°C, then washing three times with PBS and incubation with the secondary antibody at room temperature for 1 h. Thereafter, the cells were washed three times with PBS and restained with DAB, and the slides were sealed and examined under a microscope.

### 2.12. Statistical Analysis

The data results are presented as mean ± standard deviation. A one-way analysis of variance was performed to assess the between-group differences. *p* < 0.05 was deemed statistically significant, and the statistical analysis was conducted using the Statistical Program for the Social Sciences Version 18.0 (SPSS, Inc., Chicago, IL, USA). The statistical data were presented in the form of a ‘Compact Letter Display', with the symbols for the statistical analyses indicated by the letters A, B, C and D. The comparisons between the groups were considered to be statistically significant if the letters differed (*p* < 0.05) and not statistically significant if the letters were the same (*p* > 0.05).

## 3. Results

### 3.1. Antitumour Effects of SS-b2 on H22 Sarcoma Xenograft Mice

**Figure 1:**
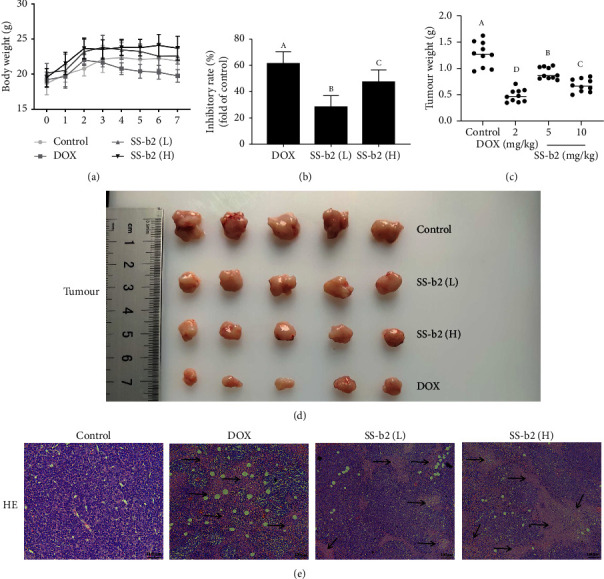
Saikosaponin-b2 (SS-b2) inhibited the tumour growth in H22 xenograft sarcoma mice. Male BALB/c mice were divided into the control group, different dose SS-b groups (5, 10 mg/kg/day) and DOX group (2 mg/kg/day) (all, *n* = 10). After 7 days of treatment, the mice were sacrificed. (a) Body weight change. (b) Tumour inhibitory rate. (c) Tumour weight. (d) Representative photographs of tumours at the end of the experiments. (e) Pathological changes in tumour tissue were observed by HE staining. The direction of the arrow is the area of structural change, with necrotic and the presence of empty spaces devoid of cells.

To ascertain the therapeutic efficacy of SS-b2 in vivo, a H22 sarcoma xenograft model was established using male mice. The mice (*n* = 10) received either vehicle (control group) or SS-b2 (5 or 10 mg/kg/day) for 7 days. Both SS-b2–treated H22 mice groups showed an inhibition of tumour growth, but no changes in body weight, when compared with the control group, suggesting a low toxicity of SS-b2 in vivo (Figure 1(a)). The average tumour weights of mice treated with SS-b2 (5 or 10 mg/kg/day) and DOX (2 mg/kg/day) were 29% ± 8.6%, 47% ± 9.5% and 62% ± 9.3% of those of control mice, respectively (Figure 1(b)). The tumour weights of the groups are shown in Figure 1(c). The tumour weights of the control group, SS-b2 (L and M) and DOX groups were 1.27 ± 0.24 g, 0.91 ± 0.11 g, 0.68 ± 0.12 g and 0.49 ± 0.12 g, respectively, indicating a significant reduction in the SS-b2 and DOX groups compared with the control group (*p* < 0.05 and *p* < 0.01, respectively). Photographs of tumours in different groups of mice are shown in Figure 1(d).

**Figure 2:**
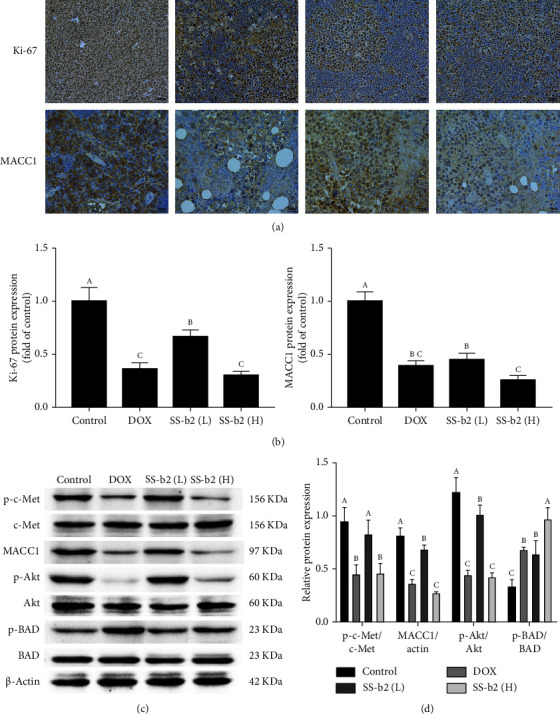
Saikosaponin b2 (SS-b2) inhibited the expression of Ki-67 and MACC1 proteins and MACC1/c-Met pathway proteins in tumour tissues in vivo. (a) Immunohistochemical staining of Ki-67 and MACC1 in xenograft tumour tissues. (b) Statistical analysis of Ki-67 and MACC1 protein expression. (c) Representative western blot photograph showing p-c-Met, MACC1, p-Akt, p-BAD or *β*-actin. (d) Statistical analysis of the MACC1/c-Met pathway–related protein expression.

For further evaluation of the pharmacokinetic activity of SS-b2 in vivo, HE immunohistochemical staining and western blotting analysis of tumour tissue were performed. The tumour tissue histology showed multipolar nucleus fission and mitosis. In contrast, SS-b2–treated tumours exhibited changes in the overall structure, including fibrosis and the presence of empty spaces devoid of cells (Figure 1(e)). In vivo proliferation was also inhibited by SS-b2 treatment, as evidenced by a reduction in Ki-67 protein levels in the tumour tissue sections of SS-b2–treated mice. Furthermore, immunohistochemical analysis demonstrated a reduction in MACC1 expression in SS-b2–treated mice compared with control mice (Figures 2(a) and 2(b)). Next, we measured the MACC1 protein levels in tumour samples and found that the in vivo treatment of SS-b2 significantly downregulated MACC1 and c-Met expression and Akt protein phosphorylation, while it enhanced the phosphorylation of BAD (Figures 2(c) and 2(d)).

### 3.2. SS-b2 Inhibited the Proliferation of HepG2 Liver Cancer Cells In Vitro

To investigate the effect of SS-b2 on the proliferation of liver cancer cell lines, the impact of varying concentrations of bupleurum SS-b2 on cellular viability after 24 h was assessed using the CCK-8 assay. As demonstrated in Table 2, SS-b2 reduced the proliferation ability of HepG2 liver cancer cells, and the effect was more obvious when the higher concentration of SS-b2 was used, which gave an IC_50_ of 0.14 mg/mL. The findings suggest that SS-b2 exerts a pronounced antiproliferative effect on HepG2 liver cancer cells.

### 3.3. SS-b2 Induced Apoptosis in HepG2 Liver Cancer Cells

**Figure 3:**
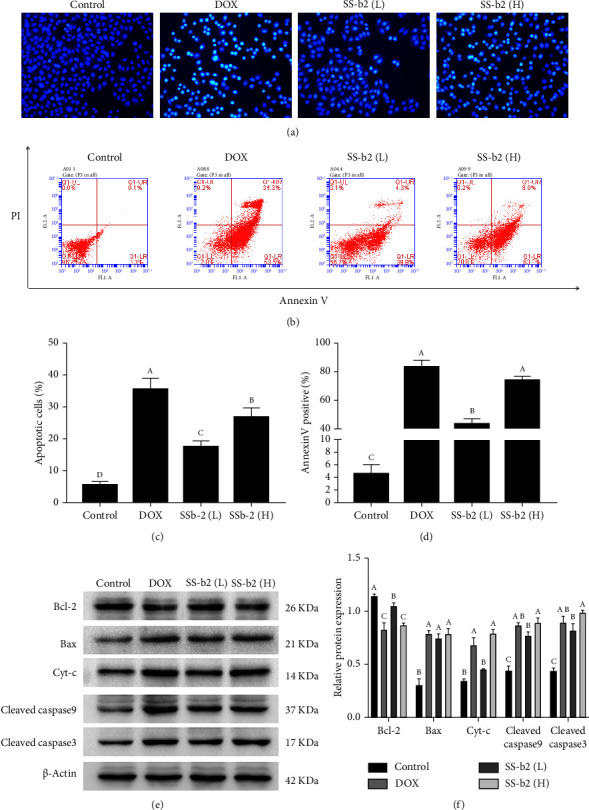
Effects of SS-b2 on HepG2 liver cancer cell apoptosis and mitochondrial apoptosis pathway–related proteins were detected. (a) HepG2 liver cancer cells were treated with doxorubicin (DOX, 2 *μ*g/mL) and SS-b2 (40 or 80 mg/L) for 24 h and then stained with Hoechst 33258. Fluorescence photomicrographs are shown (× 100). (b) The percentage of apoptosis cells from three independent experiments (c, d). After treatment with DOX and SS-b2 for 24 h, HepG2 liver cancer cells were stained with Annexin V and PI, and the apoptosis rate was detected using flow cytometry. The columns show the Annexin V positivity percentage from three independent experiments. (e) The HepG2 liver cancer cells were treated with DOX (2 *μ*g/mL) and SS-b2 (40, 80 mg/L) for 24 h and then subjected to western blot analysis. Representative western blot photograph. (f) Statistical analysis of proteins involved in the mitochondrial apoptotic pathway.

To determine whether SS-b2 can induce apoptosis in human liver cancer cells, Hoechst 33258 staining was used to assess the morphological changes in the cell nuclei via fluorescence microscopy. Typical nuclear fragments seen in SS-b2–treated cells were highly fluorescent. In contrast, control cells displayed nuclei with a normal appearance and a homogeneous blue colouration. The inhibition rates in the SS-b2 (40 and 80 mg/L) and DOX (2 *μ*g/mL) groups were 18% ± 1.8%, 27% ± 2.1% and 36 ± 3.5%, respectively (Figures 3(a) and 3(c)). Furthermore, a classical Annexin V-PI double staining was performed to quantify apoptotic cells following treatment with 40 or 80 mg/L SS-b2 for 24 h. The results of flow cytometry demonstrated a significant increase in the apoptosis rate of HepG2 liver cancer cells following the administration of SS-b2 compared with the vehicle control (Figures 3(b) and 3(d)). This indicates that the primary mode of action of SS-b2 against HepG2 liver cancer cells may be induction of apoptosis.

### 3.4. Antitumour Effect of SS-b2 by Targeting the Mitochondrial Pathway

Next, we conducted a western blot analysis to assess the impact of SS-b2 on the expression of apoptosis-associated proteins, namely, Bcl-2, Bax, Cyt-c, cleaved caspase 9 and cleaved caspase 3. Treatment with SS-b2 (40 or 80 mg/L) for 24 h caused a steady decrease in the protein expression of Bcl-2 in HepG2 liver cancer cells, whereas the expression of Bax, Cyt-c, cleaved caspase 9 and cleaved caspase 3 dramatically increased (Figures 3(e) and 3(f)). Taken together, these observations show that SS-b2 exerts its antitumour effect mainly by affecting the expression of mitochondrial pathway proteins.

### 3.5. SS-b2 Affected the Expression of MACC1/c-Met/Akt Pathway–Associated Molecules in HepG2 Liver Cancer Cells

**Figure 4:**
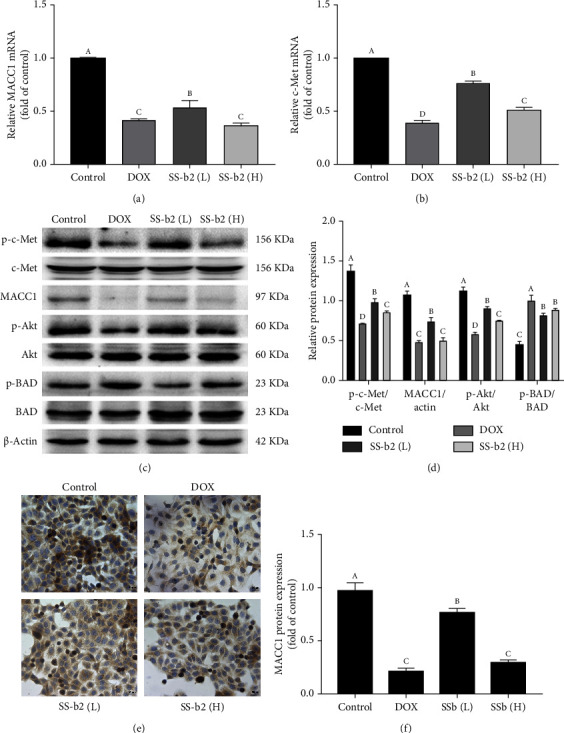
Saikosaponin-b2 (SS-b2) inhibited the mRNA and protein expression of MACC1 and c-Met in HepG2 liver cancer cells. (a, b) The mRNA expression levels of MACC1 and c-Met in HepG2 liver cancer cells treated with DOX (2 *μ*g/mL) and SS-b2 (40 or 80 mg/L) for 24 h were detected using qPCR. The mRNA expression levels were normalised to 18S rRNA. (c, d) Western blot analysis of MACC1 and c-Met expression in HepG2 liver cancer cells treated with DOX (2 *μ*g/mL) and SS-b2 (40 or 80 mg/L) for 24 h. *β*-Actin was used as a loading control. The relative protein expression of the bands was quantified and is shown in the right panel. (e, f) The protein expression of MACC1 in HepG2 liver cancer cells treated with DOX (2 *μ*g/mL) and SS-b2 (40 or 80 mg/L) for 24 h was detected by immunocytochemical staining. The integrated optical density was calculated and is shown in the right panel.

To further investigate whether the expression of MACC1/c-Met/Akt pathway–associated molecules exhibited similar changes to those above, we examined the related molecular expression by qPCR, western blotting and immunocytochemistry. The mRNA expression levels of MACC1 and c-Met in HepG2 liver cancer cells treated with different doses of SS-b2 (40 or 80 mg/L) for 24 h were significantly decreased relative to the control group (*p* < 0.05 and *p* < 0.01, respectively) (Figures 4(a) and 4(b)). SS-b2 also decreased the protein level of MACC1, downregulated the expression of phosphorescent c-Met and Akt and upregulated the expression of phosphorescent Bad relative to the control group (Figures 4(c) and 4(d)). Immunocytochemical analysis revealed that the expression of MACC1 in the cytoplasm and nucleus was reduced following the administration of SS-b2 relative to the control group (Figures 4(e) and 4(f)), in line with our qPCR and western blotting results.

**Figure 5:**
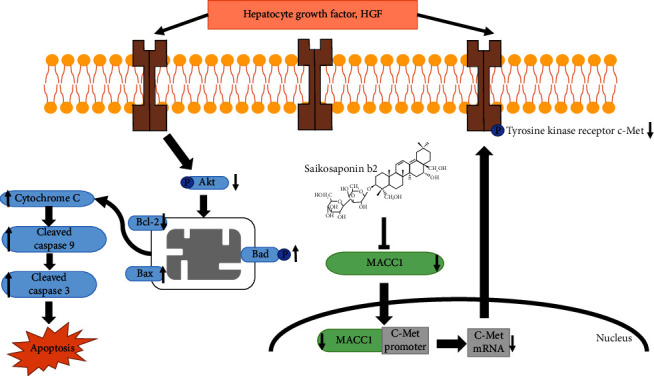
The mechanism by which saikosaponin-b2 (SS-b2) downregulates MACC1/c-Met/Akt signalling. SS-b2 reduces MACC1 levels, thereby inhibiting the phosphorylation levels of c-Met and Akt, which in turn promotes the mitochondrial apoptotic pathway. The resulting upregulation of Cyt-c, cleaved caspase 9 and cleaved caspase 3 levels ultimately led to cell apoptosis and inhibition of tumour growth.

Our results showed that SS-b2 had an obvious effect on MACC1/c-Met/Akt pathway–associated molecular expression in HepG2 liver cancer cells and H22 sarcoma xenograft mice. One possible mechanism is that MACC1, as a key regulator of the c-Met/Akt pathway, is able to suppress the expression of the transcriptional activator c-Met. In summary, these results indicated that the affection of MACC1/c-Met/Akt signalling and the inhibition of proliferation resulting from SS-b2 contribute to the repression of tumour growth in vivo (Figure 5).

## 4. Discussion

Our previous research identified the pivotal role of SS-b2 in the suppression of MACC1 in hepatocarcinogenesis and its antitumour efficacy in liver cancer. Extending our previous research, this study found that SS-b2 exerts its antitumour effects by inhibiting cell proliferation and inducing cell apoptosis through the MACC/c-Met/Akt pathway [23]. This study is the first to identify this novel mechanism of action of SS-b2 in liver cancer.

Many studies have suggested that MACC1 overexpression is associated with a range of malignant tumours in humans and with postoperative recurrence of lung adenocarcinoma. MACC1 is also highly expressed in liver cancer and is a prognostic indicator in patients with liver cancer. However, it was unknown whether MACC1 plays various biological roles in the body. This study found that MACC1 can regulate the expression and activation of the c-Met gene by binding to the gene promoter and thereby affects the c-Met signalling pathways. As a result, MACC1 promotes cell growth, epithelial–mesenchymal transition, angiogenesis, cell motility, invasiveness and metastasis through regulating the HGF/c-Met pathway [34–36]. This study found that the natural product SS-b2 exerts a pronounced suppressive effect on the protein and mRNA expression levels of MACC1. The proliferation of HepG2 liver cancer cells and of cancer cells in H22 sarcoma xenograft mice was also significantly inhibited by SS-b2, and the inhibitory effect increased with increasing SS-b2 concentration. The findings demonstrated that SS-b2 exerts an anticarcinogenic effect on the HepG2 liver cancer cells, resulting in a reduction in the expression of the antiapoptotic protein Bcl-2 and an increase in the expression of the proapoptotic protein BAD. The antitumour effects of SS-b2 suggest that proapoptosis can be initiated through the inhibition of MACC1 [23].

The objective of this study was to elucidate the mechanism by which SS-b2 inhibits MACC1 expression. To achieve this, the protein and mRNA expression of p-c-Met and MACC1 were analysed following SS-b2 treatment at varying concentrations. Our findings indicate that at low concentrations, namely, 40 and 80 *μ*g/mL, SS-b2 effectively suppressed the expression of both mRNA and protein of c-Met and MACC1 in vitro [23]. Compared to saikosaponin b2, DOX exhibits a stronger inhibitory effect on p-c-Met. An important downstream target for MACC1/c-MET signalling is PI3K/Akt signalling. Akt, also known as protein kinase B (PKB), is frequently altered in human cancers, particularly when serine/threonine protein kinase and PI3K are activated by biphosphorylation at Tyr308 and Ser473. Upon activation, Akt regulates cellular processes such as cell survival, cell cycle progression and cellular growth. In addition, activated Akt in cells contributes to the rapid dephosphorylation of BAD. The dephosphorylated BAD enters mitochondria and induces cell apoptosis. Our western blot results also showed that the expression level of p-Akt decreased, while that of p-BAD increased after SS-b2 treatment.

Studies have reported that the dysregulation of PI3K/Akt can result in the induction of mitochondria-mediated apoptosis [37]. As a central regulator of cell fate, Akt is involved in the regulation of mitochondrial apoptosis and plays a key role in the regulation of lipid metabolism in a variety of cells [37]. In this study, our most important finding was that SS-b2 treatment for a short duration decreased the tumour volumes in H22 mice without apparent adverse effects. Furthermore, a large area of cellular necrosis and karyolysis appeared in the tumour tissue, and the tumour growth was inhibited. Cell proliferation is a pivotal mechanism underlying the growth and invasion of tumours. We found that Ki-67 expression was significantly reduced in both the low- and high-dose SS-b2 groups and DOX group compared with the control group. Furthermore, SS-b2 within a specific concentration range inhibited the growth of the human liver cell line HepG2, with the inhibitory effect increasing with increasing concentration in that range. As for apoptosis, Hoechst 33258 staining showed typical changes of HepG2 liver cancer cell apoptosis after SS-b2 treatment for 24 h. This result was validated by FCM Annexin V-FITC/PI double staining, which revealed significantly higher apoptosis rates in the SS-b2 groups than in the control group. By modulating the mitochondrial pathway and the Bcl-2 family–related antiapoptotic or proapoptotic proteins, it is possible to alter the permeability of the mitochondrial outer membrane, resulting in the release of cytochrome c into the cytoplasm and activation of cleaved caspase 9 and cleaved caspase 3, which lead to apoptosis [38–40]. Bcl-2 and Bax proteins, as important prognostic biomarkers for cancer, function as a ‘molecular switch' that initiates apoptosis [41]. Our results showed that the protein expression of Bcl-2 was significantly decreased, while that of Bax, Cyt-c, cleaved caspase 9 and cleaved caspase 3 was dramatically increased, in response to SS-b2 treatment relative to the control group. These results indicate that SS-b2 inhibited the proliferation of cancer cells in H22 sarcoma xenograft mice and of HepG2 liver cancer cells by inducing apoptosis.

In conclusion, our study demonstrated that the antitumour effects of SS-b2 on liver cancer cells occur via its effect on the MACC1/c-Met/Akt pathway. Our findings suggest that SS-b2 is a promising novel candidate for the prevention and therapy of liver cancer by inhibiting the expression of MACC1. Nevertheless, our experiments have only made a preliminary exploration of its mechanism, which needs to be further verified by using blocking agents. Further investigation into the molecular mechanisms underlying the effects of SS-b2 on primary liver cancer by our study team is ongoing.

## Figures and Tables

**Table 1:** Primer sequences.

Primer name	Primer sequence (5′ to 3′)
MACC1 (F)	CCCAGGAGGCAGCTAGAAAG
MACC1 (R)	GCCTCAGACGTTCTCTCCAC
c-Met (F)	CCCGAAGTGTAAGCCCAACT
c-Met (R)	CTGCACTTGTCGGCATGAAC
18S (F)	CCTGGCACCCAGCACAAT
18S (R)	GGGCCGGACTCGTCATAC

**Table 2:** The inhibitory effect of saikosaponin-b2 on the proliferation of HepG2 liver cancer cells.

Groups	Dose (mg·L^−1^)	24 h		IC_50_ (mg·L^−1^)
		OD value	IR (%)	
Control	0	0.88 ± 0.02	—	

Saikosaponin-b2	10.0	0.86 ± 0.10	2.8	140.0 ± 3.0
	25.0	0.84 ± 0.07	5.1	
	50.0	0.80 ± 0.05	10.3	
	75.0	0.73 ± 0.04⁣^∗^	17.7	
	90.0	0.56 ± 0.05⁣^∗∗^	36.7	
	100.0	0.48 ± 0.10⁣^∗∗^	44.7	
	200.0	0.37 ± 0.04⁣^∗∗^	58.3	
	400.0	0.22 ± 0.08⁣^∗∗^	75.1	
	800.0	0.03 ± 0.02⁣^∗∗^	97.3	

*Note:* The values are expressed as mean value ± SD. *n* = 12 in each group.

⁣^∗^*p* < 0.05.

⁣^∗∗^*p* < 0.01 vs. control group.

## Data Availability

The data that support the findings of this study are available on request.
